# Experiences and attitudes of mental health care staff to the reporting of violence in the workplace in the Republic of Ireland

**DOI:** 10.1192/j.eurpsy.2022.887

**Published:** 2022-09-01

**Authors:** J. Fegan, E. Walsh

**Affiliations:** Mayo Mental Health Services, Old Age Psychiatry, Castlebar, Ireland

**Keywords:** violence, Workplace, reporting violence, mental health

## Abstract

**Introduction:**

The WHO and the Violence Prevention Alliance define violence as “the intentional use of physical force or power, threatened or actual, against oneself, another person, or against a group or community, that either results in or has a high likelihood of resulting in injury, death, psychological harm, maldevelopment, or deprivation.” The types of violence examined in this study include physical, sexual, verbal and racial as the most commonly reported manifestations of violence in the workplace.

**Objectives:**

To obtain the most recent statistics on violent acts perpetrated against mental health care workers in the Republic of Ireland. To capture the experiences and attitudes of staff to the reporting of this violence.

**Methods:**

The State Claims Agency (SCA) were contacted to obtain the most up to date figures on violence against mental health care workers. An electronic survey based on the WHO’s validated questionnaire on violence was then disseminated to all acute psychiatric units nationally.

**Results:**

There were 6,690 episodes of violence against staff in the Mental Health Division in 2018 and 2019. The survey found, 92.4% of respondents reported verbal abuse, 30.3% recorded physical assault, 15.2% had suffered sexual violence in a 24 month period. 20.3% of study participants took no action. Of those who did, 70% felt that the incident had not been investigated properly. More than half of respondents felt that there were no consequences to the aggressor.

**Conclusions:**

Further work is needed in the prevention of workplace violence as well as improvements in reporting and investigating of incidents when they do occur.

**Disclosure:**

No significant relationships.

